# Supplementary material for: Enriched sleep environments lengthen lemur sleep duration
Source: PLoS One. 2021 Nov 1;16(11):e0253251. doi: 10.1371/journal.pone.0253251 (PMC8559942; doi:10.1371/journal.pone.0253251)
Supplement: S1 File — Code and diagnostic figures from which the statistical analysis in this paper can be fully replicated. (DOCX) [file pone.0253251.s001.docx]

Effects of Sleep Site Manipulation on Lemur Sleep Quality - Statistical Report

Alexander Vining

March 10, 2021

All code and data used to generate this report can be found at <https://github.com/aqvining/Lemur_Sleep_Site_Enrichment>

# Analytical Framework

We take a step-wise approach to building and testing a series of increasingly complex linear mixed effects models to understand the processes driving twenty-four hour total sleep time (TTST) in our subject lemurs. We begin by modeling only the random effect of individual (nested within species), assuming our manipulations had no effect (model 0). We then examine the temporal autocorrelation of total sleep time across nights, choosing an autoregressive model to control for within-group correlation in our data (model 1). Finally, we add parameters describing our experimental structure to this model, including coefficients for the three levels of experimental condition, the two orders the conditions were presented in, and their interactions (model 2). We use delta AICs at each step to assess whether the more complex model provides a sufficiently improved fit to the data. Before making inferences about the effects of our experimental structure, we plot the normalized residuals against predicted values and the quantiles of a standard normal distribution to ensure our model meets the assumptions of being IID. Finally, we quantify the contrasts of each level of experimental condition, marginal to order, and test for significance in each.

# The Data

Prior to building and testing these models, we visualized TTST over time for each individual lemur

sleep_data <- read_csv("../DATA/lemur_sleep_data.csv")

## Warning: Missing column names filled in: 'X1' [1]

##
## -- Column specification --------------------------------------------------------
## cols(
## X1 = col_double(),
## Species = col_character(),
## Sociality = col_character(),
## Nestbuild = col_character(),
## Activity = col_character(),
## study.day = col_double(),
## Experiment = col_character(),
## ID = col_character(),
## dayvars.counts = col_double(),
## dayvars.cpm = col_double(),
## TTST = col_double(),
## night.cpm = col_double(),
## day.from.zero = col_double(),
## Session = col_double()
## )

sleep_data$Session <- factor(sleep_data$Session)
sleep_data$Species <- factor(sleep_data$Species)
sleep_data$Experiment <- factor(sleep_data$Experiment, levels = c("base", "absence", "enhance")) #order factor levels of experimental condition to set up baseline as intercept
sleep_data$ID <- factor(sleep_data$ID, levels = c("Aria", "Geb", "Bertha", "Beatrice", "Avior", "Josephine", "Persephone", "Teres")) #order factor levels for nice plots

#add the experimental order to data frame (previously encoded trial by trial in the Session column)
sleep_data$Order <- rep(factor(c("absence", "enhance", "absence", "enhance", "absence", "enhance", "absence", "enhance")),
 times = sapply(levels(sleep_data$ID), function(X) nrow(filter(sleep_data, ID == X))))

## # A tibble: 8 x 10
## ID Base_N Base_Mean Base_sd Enriched_N Enr_Mean Enr_sd Impoverished_N
## <fct> <int> <dbl> <dbl> <int> <dbl> <dbl> <int>
## 1 Aria 6 560. 32.4 7 563. 41.7 7
## 2 Geb 6 643. 25.8 7 642. 56.1 7
## 3 Bertha 7 729. 47.5 7 759. 35.2 7
## 4 Beatrice 7 658. 48.2 7 721. 50.9 6
## 5 Avior 7 626. 51.1 7 666. 42.7 7
## 6 Josephine 7 601. 34.5 7 636 41.2 7
## 7 Persephone 6 568. 18.8 7 612. 28.2 7
## 8 Teres 6 731 34.9 7 770. 49.5 7
## # ... with 2 more variables: Imp_Mean <dbl>, Imp_sd <dbl>


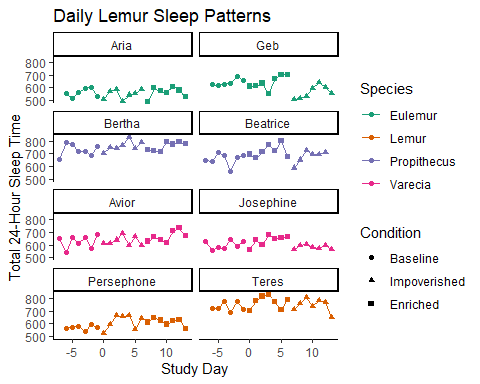


## png
## 2

# Null Model: Random Effects

We begin simply by modeling our data as normally distributed around a mean, with random effects to that mean for each individual (nested within its species). Anticipating autoregressive effects because these data are a time-series of a biorhythm, we visuallize the autocorrelation up to the duration of a single experimental condition.

m0_lemur_sleep <- lme(TTST ~ 1, data = sleep_data, random = ~1|Species/ID, method = "ML")
plot(ACF(m0_lemur_sleep, maxLag = 6), alpha = 0.01)


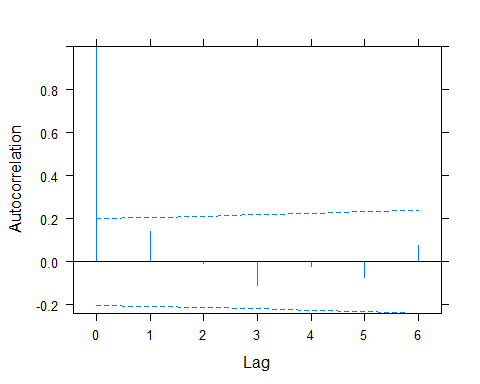


We do not see significant autocorrelation, however, because of our strong prior expectations that these time series data have some temporal dependencies we chose to implement temporal structure into our model anyway, but based on these plots start with a simple AR structure.

# Model 1: Time-Series Analysis

We thus modeled TTST as a first order auto-regressive process (AR1) centered on some true mean with individual-based variance in this mean plus noise. In non-jargon terms, this means we predicted any given lemur’s sleep on any given night based on an estimated mean TTST for that lemur, that lemur’s deviance from their mean TTST on the previous two nights, and an estimation of observation error across all lemurs and nights. Formally

$$TTST_{s,j,t}=\mu+\phi_{1}(TTST_{s,j,t-1})+U_{s}+V_{j}+E_{s,j,t}$$

where $TTST_{s,j,t}$ is the predicted TTST of individual $j$ from species $s$ during day $t$, $\beta_{1}$ is the intercept (mean) of TTST, $\phi_{1}$ is the magnitude of the first order temporal auto-regression, $U_{s}$ is the random effect of species $s$, $V_{j}$ is the random effect of individual $j$, and $E_{s,j,t}$ is an error term, for which all values are assumed to be normally distributed about 0.

m1_lemur_sleep <- update(m0_lemur_sleep, correlation = corARMA(p = 1, form = ~ day.from.zero | Species/ID), method = "ML")
summary(m1_lemur_sleep)

## Linear mixed-effects model fit by maximum likelihood
## Data: sleep_data
## AIC BIC logLik
## 1747.109 1762.578 -868.5546
##
## Random effects:
## Formula: ~1 | Species
## (Intercept)
## StdDev: 23.21154
##
## Formula: ~1 | ID %in% Species
## (Intercept) Residual
## StdDev: 60.97084 46.94578
##
## Correlation Structure: AR(1)
## Formula: ~day.from.zero | Species/ID
## Parameter estimate(s):
## Phi
## 0.2058285
## Fixed effects: TTST ~ 1
## Value Std.Error DF t-value p-value
## (Intercept) 650.1834 24.96454 155 26.04428 0
##
## Standardized Within-Group Residuals:
## Min Q1 Med Q3 Max
## -2.60278657 -0.67493319 0.05370719 0.65085234 2.53079470
##
## Number of Observations: 163
## Number of Groups:
## Species ID %in% Species
## 4 8

plot(ACF(m1_lemur_sleep, maxLag = 6, resType = "n"), alpha = 0.01) #reduced lag one correlation of normalized residuals. Still notable correlation at lag 9, but given this high order, non-significance, and small dataset, probably not worth worrying about.


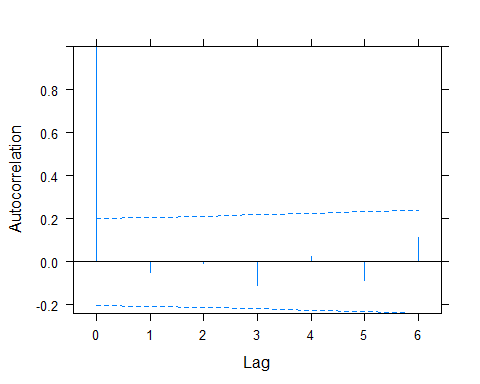


AIC(m0_lemur_sleep, m1_lemur_sleep)

## df AIC
## m0_lemur_sleep 4 1750.985
## m1_lemur_sleep 5 1747.109

The delta AIC between model 1 and model 0 validates the inclusion of an AR1 term, and our ACF plots suggest we have sufficiently controlled for within-group data correlation to not violate IID assumption in further models.

# Model 2: Experimental Structure

Having chosen a within-group correlation structure, we next build our experimental structure into our model. Here, we must simultaneously account for the possible effects of our experimental conditions, the order they were presented in, and interactions between the condition and the order (eg crossover or carryover effects). We achieve this with the following equation.

$$TTST_{s,j,t}=\mu+\phi_{1}(y_{s,j,t-1})+\beta_{C}Condition_{s,j,t}+\beta_{O}Order_{s,j}+\beta_{C,O}Condition_{s,j,t}*Order_{s,j}+U_{s}+V_{j}+E_{s,j,t}$$

where $\beta_{C}$ is the regression coefficient for the experimental condition given by $Condition_{s,j,t}$, $\beta_{O}$ is the regression coefficient for order of experimental conditions given by $Order_{s,j}$, $\beta_{C,O}$ is the regression coefficient for the interaction of the condition-order pair given by $Condition_{s,j,t}*Order_{s,j}$, and all other terms are as in Equation 1.

m2_lemur_sleep <- update(m1_lemur_sleep, . ~ Experiment * Order, method = "ML")
summary(m2_lemur_sleep)

## Linear mixed-effects model fit by maximum likelihood
## Data: sleep_data
## AIC BIC logLik
## 1742.429 1773.366 -861.2145
##
## Random effects:
## Formula: ~1 | Species
## (Intercept)
## StdDev: 15.01399
##
## Formula: ~1 | ID %in% Species
## (Intercept) Residual
## StdDev: 66.53319 43.6428
##
## Correlation Structure: AR(1)
## Formula: ~day.from.zero | Species/ID
## Parameter estimate(s):
## Phi
## 0.0886447
## Fixed effects: TTST ~ Experiment + Order + Experiment:Order
## Value Std.Error DF t-value p-value
## (Intercept) 639.6882 27.42262 150 23.327031 0.0000
## Experimentabsence 11.9661 13.21587 150 0.905435 0.3667
## Experimentenhance 33.9458 13.15279 150 2.580884 0.0108
## Orderenhance 0.0677 18.06518 150 0.003749 0.9970
## Experimentabsence:Orderenhance -22.6012 19.04436 150 -1.186765 0.2372
## Experimentenhance:Orderenhance -5.9010 18.96478 150 -0.311155 0.7561
## Correlation:
## (Intr) Exprmntb Exprmntn Ordrnh Exprmntb:O
## Experimentabsence -0.181
## Experimentenhance -0.183 0.527
## Orderenhance -0.316 0.192 0.195
## Experimentabsence:Orderenhance 0.096 -0.715 -0.387 -0.307
## Experimentenhance:Orderenhance 0.098 -0.387 -0.715 -0.309 0.549
##
## Standardized Within-Group Residuals:
## Min Q1 Med Q3 Max
## -2.4558865 -0.5936132 0.1150436 0.6995556 2.3761249
##
## Number of Observations: 163
## Number of Groups:
## Species ID %in% Species
## 4 8

plot(ACF(m2_lemur_sleep, maxLag = 5, resType = "n"), alpha = 0.01)


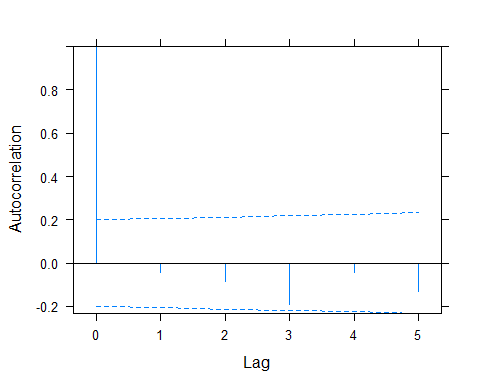


AIC(m2_lemur_sleep,m1_lemur_sleep) #despite introducing some lag3 autocorrelation into within-group normalized residuals, experimental structure greatly increases the model fit.

## df AIC
## m2_lemur_sleep 10 1742.429
## m1_lemur_sleep 5 1747.109

intervals(m2_lemur_sleep)

## Approximate 95% confidence intervals
##
## Fixed effects:
## lower est. upper
## (Intercept) 586.510311 639.68819330 692.86608
## Experimentabsence -13.662072 11.96611594 37.59430
## Experimentenhance 8.439969 33.94584204 59.45172
## Orderenhance -34.964232 0.06773153 35.09970
## Experimentabsence:Orderenhance -59.531961 -22.60118229 14.32960
## Experimentenhance:Orderenhance -42.677460 -5.90098925 30.87548
## attr(,"label")
## [1] "Fixed effects:"
##
## Random Effects:
## Level: Species
## lower est. upper
## sd((Intercept)) 0.0003353245 15.01399 672244.3
## Level: ID
## lower est. upper
## sd((Intercept)) 31.09784 66.53319 142.3464
##
## Correlation structure:
## lower est. upper
## Phi -0.08314598 0.0886447 0.2553188
## attr(,"label")
## [1] "Correlation structure:"
##
## Within-group standard error:
## lower est. upper
## 38.93989 43.64280 48.91370

Satisfied that we are justified in included experimental structure in the data, we assess the asumptions of our model by looking at standardized residuals.

diagnostics_data <- mutate(m2_lemur_sleep$data, prediction = predict(m2_lemur_sleep), residual = residuals(m2_lemur_sleep))
ggplot(diagnostics_data) + geom_point(aes(x = prediction, y = residual/sd(residual))) + theme_classic() #Pearson's residuals on y. largely normal and homoskedastic


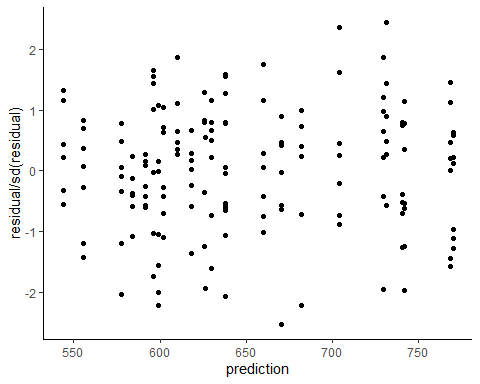


qqnorm(m2_lemur_sleep) #pearson residual quantiles look standard normal


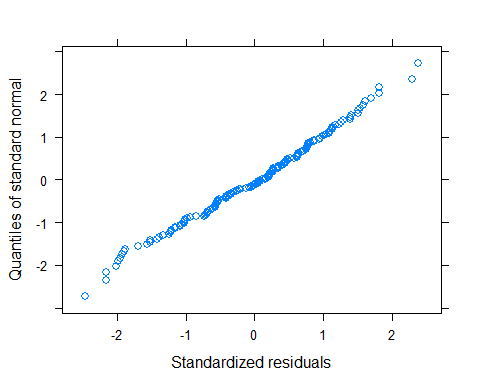


All assumptions of normality, homoskedasticity, and stationarity look valid.

# Statistical Inference

Because order effects and their interaction with experimental condition could potentially confound an analysis that examines experimental condition only, we use our estimated model parameters to calculate the contrast of each experimental condition relative to the others, marginal to order. Noting that the intercept represents baseline sleep when the impoverishment condition was presented before the enrichment, we calculate marginalized contrasts as in the code below.

#effect contrasts marginal to order, get means and se

#manual calculation of mean contrast of enriched vs. impoverished; used to validate matrix multiplication methods useful for attaining contrast se, as done further below
experiment_contrast_marginal_order <- m2_lemur_sleep$coefficients$fixed["Experimentenhance"] - m2_lemur_sleep$coefficients$fixed["Experimentabsence"] + diff(m2_lemur_sleep$coefficients$fixed[c("Experimentabsence:Orderenhance","Experimentenhance:Orderenhance")])/2 #diff subtracts first element from second

##~enhanced v baseline
enhanced_v_baseline_algebra <- c(intercept = 0, impoverish = 0, enrich = 1, enrich_first = 0, imp_by_enr1st = 0, enr_by_enr1 = 0.5) #When multiplied by coefficients matrix from model 2, calculates effect of enrichment marginal to order (with equal weights)
enhanced_v_baseline_contrast <- enhanced_v_baseline_algebra %*% m2_lemur_sleep$coefficients$fixed
enhanced_v_baseline_contrast_se <- t(enhanced_v_baseline_algebra) %*% vcov(m2_lemur_sleep) %*% enhanced_v_baseline_algebra %>% #quadratic form to evaluate the variance of a contrast.sqrt(enhanced_v_absence_contrast_var)
 sqrt()

##~absence v baseline
absence_v_baseline_algebra <- c(intercept = 0, impoverish = 1, enrich = 0, enrich_first = 0, imp_by_enr1st = 0.5, enr_by_enr1 = 0)#When multiplied by coefficients matrix from model 2, calculates effect of impoverishment marginal to order (with equal weights)
absence_v_baseline_contrast <- absence_v_baseline_algebra %*% m2_lemur_sleep$coefficients$fixed
absence_v_baseline_contrast_se <- t(absence_v_baseline_algebra) %*% vcov(m2_lemur_sleep) %*% absence_v_baseline_algebra %>% #quadratic form to evaluate the variance of a contrast.sqrt(enhanced_v_absence_contrast_var)
 sqrt()

##~enhanced v absence
enhanced_v_absence_algebra <- c(intercept = 0, impoverish = -1, enrich = 1, enrich_first = 0, imp_by_enr1st = -0.5, enr_by_enr1 = 0.5) #rename algebra. When multiplied by coefficients from model 2, does the algebra from line 86. Can also be multiplied by vcv to find se.
enhanced_v_absence_contrast <- enhanced_v_absence_algebra %*% m2_lemur_sleep$coefficients$fixed #equivalent to line 86
enhanced_v_absence_contrast_se <- t(enhanced_v_absence_algebra) %*% vcov(m2_lemur_sleep) %*% enhanced_v_absence_algebra %>% #quadratic form to evaluate the variance of a contrast.sqrt(enhanced_v_absence_contrast_var)
 sqrt()


print(paste("contrast of enriched and baseline sleep marginal to order is", enhanced_v_baseline_contrast, "with se = ", enhanced_v_baseline_contrast_se, sep = " "))

## [1] "contrast of enriched and baseline sleep marginal to order is 30.9953474171772 with se = 9.02555919525292"

print(paste("contrast of impoverished and baseline sleep marginal to order is", absence_v_baseline_contrast, "with se = ", absence_v_baseline_contrast_se, sep = " "))

## [1] "contrast of impoverished and baseline sleep marginal to order is 0.665524798907345 with se = 9.06784888480797"

print(paste("contrast of enriched and absence sleep marginal to order is", enhanced_v_absence_contrast, "with se = ", enhanced_v_absence_contrast_se, sep = " "))

## [1] "contrast of enriched and absence sleep marginal to order is 30.3298226182699 with se = 8.85787465563119"

contrast_matrix <- matrix(c(enhanced_v_baseline_algebra, absence_v_baseline_algebra, enhanced_v_absence_algebra), nrow = 3, byrow = TRUE, dimnames = list(c("enrich_base", "impoverish_base", "enrich_impoverish"),names(m2_lemur_sleep$coefficients$fixed)))
contrast_matrix

## (Intercept) Experimentabsence Experimentenhance Orderenhance
## enrich_base 0 0 1 0
## impoverish_base 0 1 0 0
## enrich_impoverish 0 -1 1 0
## Experimentabsence:Orderenhance Experimentenhance:Orderenhance
## enrich_base 0.0 0.5
## impoverish_base 0.5 0.0
## enrich_impoverish -0.5 0.5

anova(m2_lemur_sleep, L = contrast_matrix[1,]) #baseline v enriched contrast, significantly different

## F-test for linear combination(s)
## Experimentenhance Experimentenhance:Orderenhance
## 1.0 0.5
## numDF denDF F-value p-value
## 1 1 150 11.35944 0.001

anova(m2_lemur_sleep, L = contrast_matrix[2,]) #baseline v impoverished, not significantly different

## F-test for linear combination(s)
## Experimentabsence Experimentabsence:Orderenhance
## 1.0 0.5
## numDF denDF F-value p-value
## 1 1 150 0.005188382 0.9427

Correcting our significance level to 0.167 using a Bonferroni adjusment for three tests, we can reject the hypotheses that sleep site enrichment does not affect total sleep time relative to baseline and impoverished conditions, but we fail to reject the hypothesis that sleep site impoverishment does not affect total sleep times relative to baseline.

# Random Effects

We calculate intra-class correlation coefficients to compare the unstructured variance in our data attributable to each of our random effects.

#Compare sources of variance
var_cov_rand <- VarCorr(m2_lemur_sleep) #variance covaraince matrix of random effects in model 2
suppressWarnings(storage.mode(var_cov_rand) <- "numeric") #VarCorr returns a character table, this converts to numeric without messing up the structure

ICC_Species <- var_cov_rand[2, "Variance"]/(sum(var_cov_rand[,"Variance"], na.rm = TRUE))
ICC_ID <- var_cov_rand[4, "Variance"]/(sum(var_cov_rand[,"Variance"], na.rm = TRUE))
ICC_Residual <- var_cov_rand[5, "Variance"]/(sum(var_cov_rand[,"Variance"], na.rm = TRUE))
ICC_Species

## [1] 0.03437968

ICC_ID

## [1] 0.675128

ICC_Residual

## [1] 0.2904923
