# Supplementary material for: Enriched sleep environments lengthen lemur sleep duration
Source: PLoS One. 2021 Nov 1;16(11):e0253251. doi: 10.1371/journal.pone.0253251 (PMC8559942; doi:10.1371/journal.pone.0253251)
Supplement: Attachment — Submitted filename: Response to Reviewers_Lemur_Sleep2.docx [file pone.0253251.s003.docx]

**Enriched sleep environments lengthen lemur sleep duration – Response to Reviewers**

Dear Dr. Vyazovskiy,

Thank you for you continued support in publishing this manuscript. We believe that the changes we have made are sufficient to address the remaining reviewer concerns. Those changes are detailed below, comment by comment.

1. “One issue that has not been fully addressed yet is that recordings of rest-activity patterns are only a correlate of true sleep-wake patterns (mentioned under my previous point 1). In other words, the authors equate a lack of movement as measured with motion watches with sleep. However, it is not excluded that differences in the the amount of movement between conditions reflect, perhaps partly, differences in relaxed wakefulness rather than differences in true sleep. The lemurs in the enriched condition with the high quality sleeping-box, lined with comfortable soft bedding may just be more inclined to stay in their nest a bit longer, even when they are awake. I feel this is not a trivial point, given that the differences in rest and/or sleep between the enriched and impoverished condition amounts to not much more than 30 min on average.”

We would like to thank the reviewer for their concern, and agree that we should be as transparent as possible about what we measured. Though we think that our inferential criteria for categorizing sleep are quite conservative and unlikely to capture much restful wakefulness, we have added a few sentences to make clear to our readers that it is a possibility. First, we added a sentence to the methods (line 157) explaining the possibility that we may be capturing wakefulness in our quantitative definition of sleep. Additionally, we now highlight in the first paragraph of our discussion (line 228) that using body-motion as a proxy for sleep limits our ability to make conclusions about specific sleep states such as REM of SWS.

2. Also, with the previous points in mind, I have mild concerns about the use of the wording 'strong evidence' in both the abstract (line 37) and discussion (line 316). They may want to tone down a little bit.

Thank you for the note. We removed “strong” from both of these sentences.

Best regards,

Alexander, Charlie, and David.

October 4, 2021
